# Challenges and Opportunities of Choosing a Membrane for Electrochemical CO_2_ Reduction

**DOI:** 10.3390/membranes15020055

**Published:** 2025-02-08

**Authors:** Helene Rehberger, Mohammad Rezaei, Abdalaziz Aljabour

**Affiliations:** GIG Karasek GmbH, Neusiedlerstraße 15-19, A-2640 Gloggnitz, Austria; h.rehberger@gigkarasek.at (H.R.); a.aljabour@gigkarasek.at (A.A.)

**Keywords:** electrochemical CO_2_ reduction, carbon dioxide, carbon monoxide, syngas, CO_2_ emissions, ion exchange membrane, cation exchange membrane, anion exchange membrane, bipolar membrane

## Abstract

The urgent need to reduce greenhouse gas emissions, particularly carbon dioxide (CO_2_), has led to intensive research into novel techniques for synthesizing valuable chemicals that address climate change. One technique that is becoming increasingly important is the electrochemical reduction of CO_2_ to produce carbon monoxide (CO), an important feedstock for various industrial processes. This comprehensive review examines the latest developments in CO_2_ electroreduction, focusing on mechanisms, catalysts, reaction pathways, and optimization strategies to enhance CO production efficiency. A particular emphasis is placed on the role of ion exchange membranes, including cation exchange membranes (CEMs), anion exchange membranes (AEMs), and bipolar membranes (BPMs). The review explores their advantages, disadvantages, and the current challenges associated with their implementation in CO_2_ electroreduction systems. Through careful analysis of the current literature, this report aims to provide a comprehensive understanding of state-of-the-art methods and their potential impact on sustainable CO production, with a special focus on membrane technologies.

## 1. Introduction

The 21st century presents humanity with unprecedented environmental challenges, foremost among which is the urgent need to reduce greenhouse gas emissions in order to combat climate change. Among these emissions, carbon dioxide (CO_2_) stands out as one of the main contributors to global warming. The continued reliance on fossil fuels for energy production, transportation, and industrial processes has led to a steady increase in CO_2_ concentrations in the atmosphere and has exacerbated the climate crisis [1].

**Figure 1:**
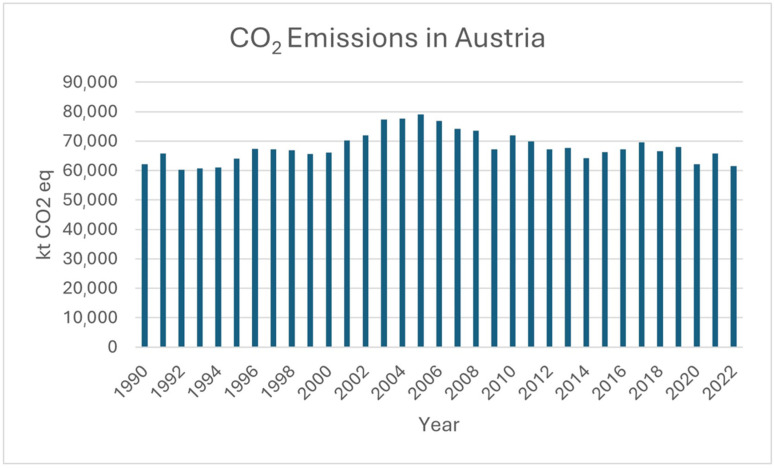
CO_2_ Emissions in Austria from 1990 to 2022 [1].

In Figure 1, the CO_2_ emissions in Austria from 1990 to 2022 are illustrated. To address this amount of emitted CO_2_, researchers have been exploring innovative strategies to reduce CO_2_ emissions while meeting energy and industrial needs. Carbon capture utilization (CCU) has proven to be a promising approach to reduce CO_2_ emissions by converting captured CO_2_ into valuable products. In the context of CCU, electrochemical reduction of CO_2_ is particularly promising as it can convert CO_2_ into useful chemicals and fuels, thus, closing the carbon cycle and contributing to a circular economy [2].

In this review, the concept of carbon electroreduction is introduced as a key strategy within the broader CCU framework. We outline the motivations driving research in this area, including the need to decarbonize the economy, diversify energy sources, and promote sustainable development. By examining the role of CO_2_ electricity reduction in achieving climate change goals, we set the stage for a comprehensive exploration of the latest advances in this area [2].

### 1.1. Carbon Capture Utilization (CCU)

CCU is a promising approach to reducing CO_2_ emissions while producing valuable products. CCU represents a paradigm shift in the approach to reducing CO_2_ emissions by focusing on converting captured CO_2_ into useful products instead of simply storing it underground. By utilizing CO_2_ as a feedstock for chemical synthesis and industrial processes, CCU offers a double benefit: it reduces greenhouse gas emissions while creating economic value [2,3].

In the field of CCU, electrochemical CO_2_ reduction has gained significant attention due to its versatility and efficiency in converting CO_2_ into a range of valuable chemicals and fuels. Unlike traditional carbon capture and storage (CCS) methods, which capture and sequester CO_2_ emissions, electrochemical CO_2_ reduction enables the conversion of CO_2_ into products such as carbon monoxide (CO), methane (CH_4_), ethylene (C_2_H_4_) and formic acid (HCOOH) [4].

Potential applications of CO as a feedstock for various industrial processes including metal production, hydrogen generation, and chemical synthesis. Carbon monoxide (CO) serves as a versatile building block for numerous industrial processes, ranging from metal production to chemical synthesis and hydrogen generation. As a feedstock, CO finds applications in a wide range of sectors, including petrochemicals, pharmaceuticals, and materials science. The electrochemical reduction of carbon dioxide (CO_2_) offers a sustainable pathway for CO production, enabling the synthesis of CO from renewable electricity and captured CO_2_ emissions [5].

In metal production, CO serves as a reducing agent in processes such as iron smelting and steelmaking, where it reacts with metal oxides to yield pure metals. In chemical synthesis, CO is used as a precursor to produce a variety of organic compounds, including alcohols, acids, and esters. In hydrogen generation, CO can be converted into hydrogen gas via the water–gas shift reaction, providing a clean and renewable source of hydrogen for fuel cells and industrial applications [5].

Furthermore, CO finds applications as fuel and chemical feedstock in the synthesis of methanol, ammonia, and other valuable chemicals. By harnessing CO as a platform molecule, it is possible to create a wide range of products with diverse applications in industry, transportation, and energy storage. The sustainable production of CO via electrochemical CO_2_ reduction offers a promising pathway for meeting industrial demands while reducing greenhouse gas emissions and promoting a circular economy [5].

### 1.2. Conventional Strategies for CO Production

The production of carbon monoxide (CO) is an essential part of various industrial processes, including the synthesis of chemicals, fuels, and materials. In the past, CO was mainly produced by conventional processes such as coal gasification, steam reforming of natural gas, and partial oxidation of hydrocarbons. These processes are based on high-temperature processes for the thermal decomposition of carbonaceous feedstocks, producing CO together with other by-products [6].

In coal gasification, one of the oldest methods of CO production, coal is converted into a gas mixture of CO, hydrogen (H_2_), and other gasses through high-temperature reactions in the presence of steam or oxygen. Similarly, steam reforming of natural gas uses steam and a catalyst to react with methane (CH_4_) to produce CO and H_2_. In partial oxidation, hydrocarbons are burned with a limited supply of oxygen, producing CO and H_2_ [6].

While these conventional methods must fulfill the industry’s CO production needs, they have several drawbacks, such as high energy consumption, greenhouse gas emissions, and dependence on fossil fuels. As the global transition to sustainable energy and production intensifies, there is a growing need to explore alternative ways to produce CO that are more efficient, environmentally friendly, and economically viable [6].

## 2. Novel Green Conversion of CO_2_ into CO

With the growing urgency to mitigate climate change, researchers are increasingly turning to new technologies to convert carbon dioxide (CO_2_) into valuable chemicals and fuels. Among these technologies, electrochemical CO_2_ reduction holds promise because of its ability to selectively convert CO_2_ into carbon monoxide (CO) using renewable electricity as the driving force. By harnessing the power of electrons, electrochemical CO_2_ reduction offers a sustainable way to produce CO.

**Figure 2:**
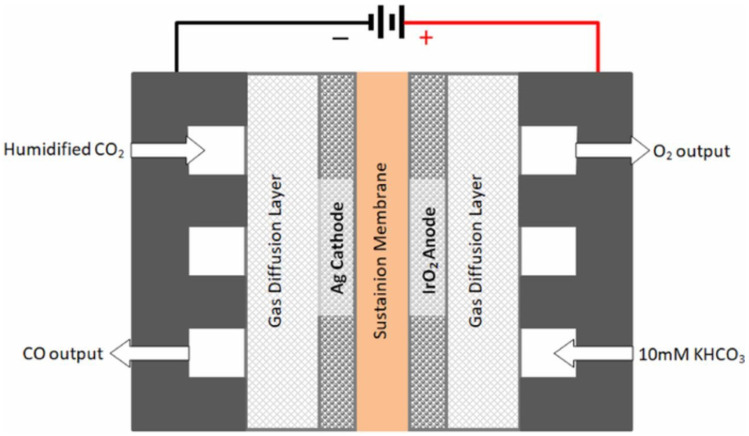
Principle of CO_2_ electrolyzer. Reprinted with permission from Ref. [7]. Copyright The Author(s) 2018. Published by ECS.

The general technical scheme of the CO_2_ electroreduction cell is illustrated by Figure 2 [7]. The electrochemical cell is a membrane electrode assembly. The Ag-based cathode represents the catalyst, which can be varied in order to produce CO. In this setup, an aqueous solution is used as the electrolyte. In the setup, the aqueous solution is 10 mM KHCO_3_.

By improving the electrochemical CO_2_ reduction to CO, a syngas production route is established, which is independent on fossil fuels [8]. In Table 1, a comparison based on current research and literature in the field of CO_2_ reduction technologies is summarized. In general, the products are generated at the cathode side via the electrochemical CO_2_ reduction and the H_2_O is oxidized at the anode side.

## 3. Electrochemical CO_2_ Reduction Mechanisms

In-depth analysis of electrochemical reduction pathways for the conversion of CO_2_ to CO is needed.

The electrochemical reduction of carbon dioxide (CO_2_) offers a promising route for the conversion to carbon monoxide (CO), a valuable industrial raw material, while reducing CO_2_ emissions. Central to this process is the electrochemical reactions that take place at the electrode surface and drive the conversion of CO_2_ to CO via a series of intermediate steps. Understanding the mechanisms underlying these reactions is crucial for optimizing the efficiency and selectivity of CO production [11].

**Figure 3:**
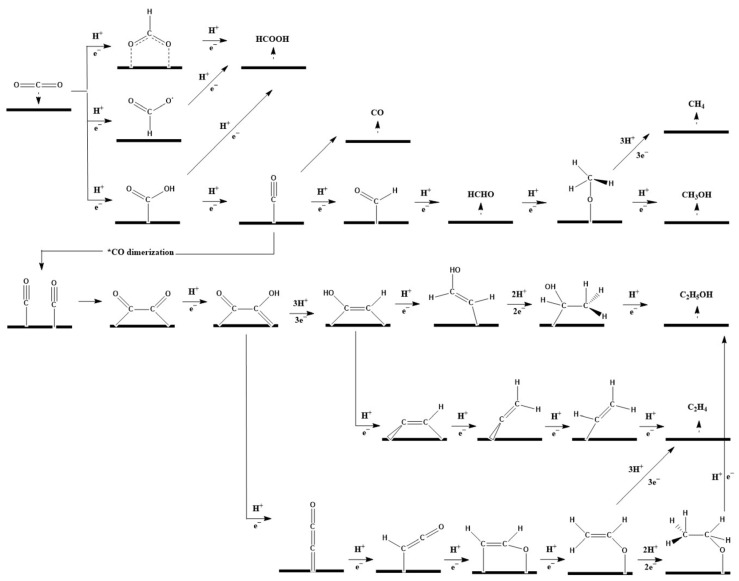
The proposed mechanism on electrochemical CO_2_ reduction. Reprinted with permission from Ref. [12]. Copyright 2022 Lin, J., Yan, S., Zhang, C., Hu, Q., & Cheng, Z.

The electrochemical reduction of CO_2_ usually proceeds via several pathways, each comprising a sequence of elementary steps that determine the formation of specific products. These pathways are influenced by factors such as the electrode material, the reaction conditions, and the composition of the electrolyte, which determine the kinetics and thermodynamics of the electrochemical process. The main intermediates of the CO_2_ reduction mechanism include adsorbed CO_2_ species, CO_2_ ions, and various surface-bound species formed during the reduction process (see Figure 3) [11].

- Adsorption strength for the key intermediate (∗COOH);
- proper CO adsorption energy;
- inert CO reduction ability;
- abundant and inexpensive raw materials;
- efficient production of H_2_ and CO simultaneously.

## 4. Catalysts for Electrochemical CO_2_ Reduction

**Figure 4:**
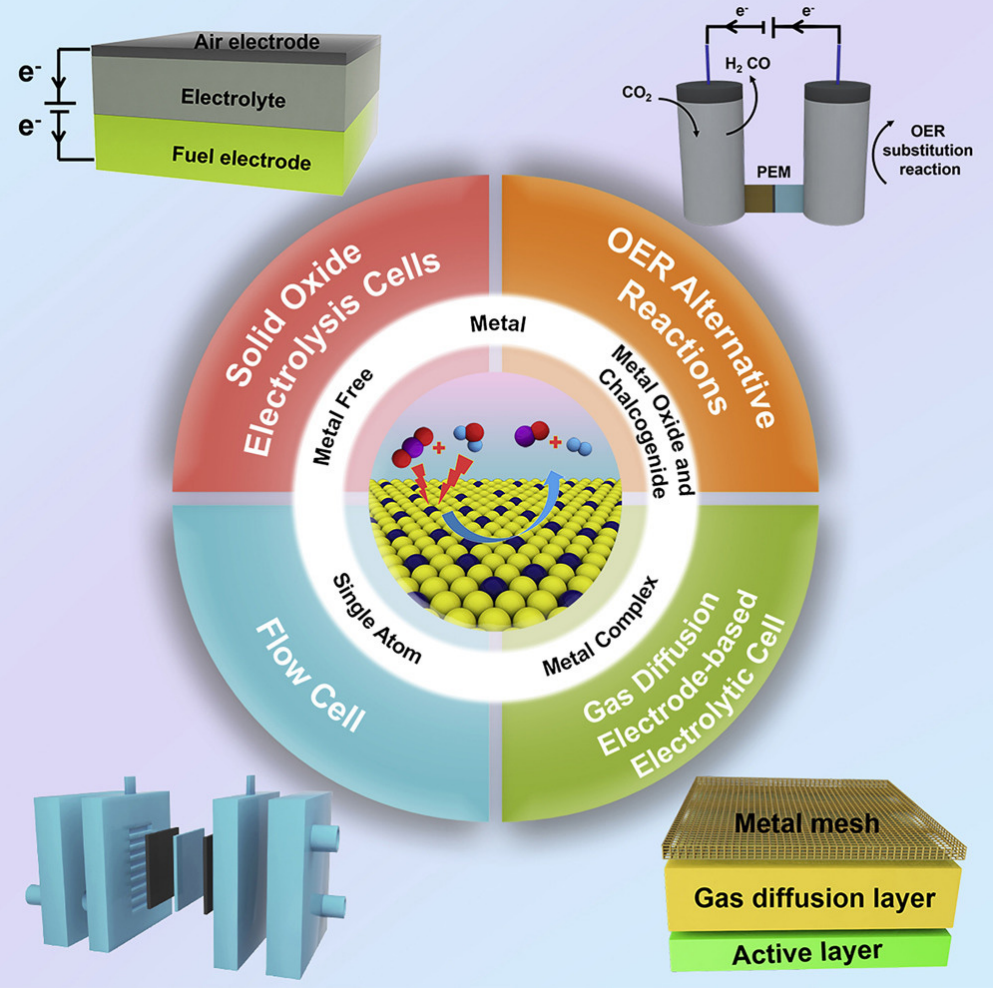
Types of electrocatalysts. Reprinted with permission from Ref. [13]. Copyright© 2020 The Author(s).

There are different types of catalysts, including metals, metal oxides, chalcogenides, metal complexes, monatomic catalysts, and metal-free catalysts, which is illustrated in Figure 4 [13].

Catalysts play a crucial role in the electrochemical reduction of carbon dioxide (CO_2_) by facilitating the conversion of CO_2_ into valuable products such as carbon monoxide (CO), methane (CH_4_) and ethylene (C_2_H_4_). A wide range of catalysts has been explored for CO_2_ electroreduction, including metals, metal oxides, chalcogenides, metal complexes, single-atom catalysts and metal-free materials. Each type of catalyst offers unique advantages and challenges in terms of activity, selectivity, stability, and cost effectiveness [13].

In addition to inorganic catalysts, organic molecules and complexes have also been explored as catalysts for CO_2_ electroreduction. Metal complexes containing transition metals such as iron (Fe), cobalt (Co) and nickel (Ni) show catalytic activity in CO_2_ reduction, while single-atom catalysts provide precise control over active sites and reaction pathways. According to the literature [14], the total reaction barrier for an Fe surface suggests a high activity on the Fe surface. The reaction barrier matches with the reaction energies as Fe < Co < Ni < Cu. Additionally, metal-free catalysts based on carbon materials, nitrogen-doped graphene, and other carbonaceous substrates are promising for CO_2_ electroreduction in aqueous and non-aqueous electrolytes [15].

As an example, copper is a useful non-noble metal catalyst, which is able to produce hydrocarbons as well as alcohols through the reduction of CO_2_ [16]. It is important to mention that by using copper a high overpotential is needed and, therefore, it influences energy efficiency negatively. The performance of Cu can be improved by changing the size and morphology design, alloying, as well as surface oxidation–reduction [16,17,18,19,20,21].

In order to improve the performance, nanoparticles (NP) provide a large surface area as well as active sites with a low coordination number such as surface planes, edges, and corners. For instance, by using diameters of around 7 nm, a faradaic efficiency of 76% of methane is obtained, which is higher than compared with the result of polycrystalline Cu, which is only 44% of faradaic efficiency. The trend shows that a smaller Cu-NP size enables a larger total current density. However, it generates lower selectivity in terms of hydrocarbon formation [19].

Table 2 shows a summary of Cu-based catalysts, tested to determine the faradaic efficiency as well as the current density [20].

**Figure 5:**
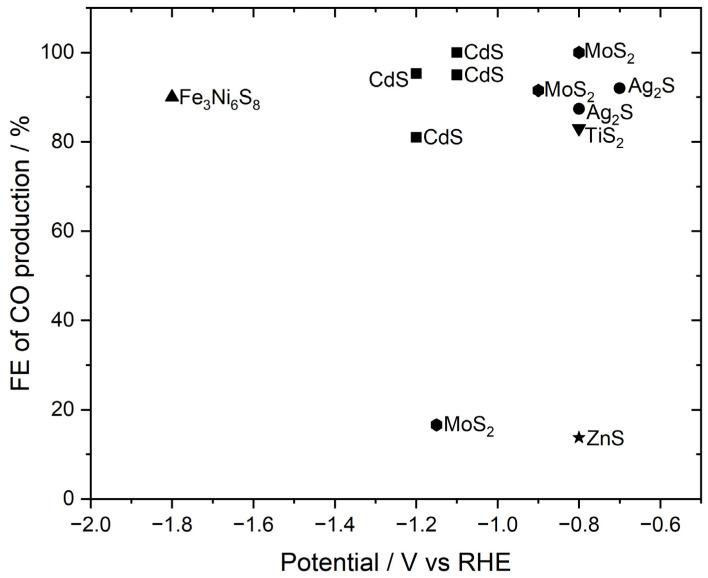
Summary of CO2RR results reported in the literature [18,22,23,24,25,26,27,28,29,30,31,32,33].

In order to produce CO, there are many inorganic-based catalysts. By changing the potential different faradaic efficiencies are reported in the literature. The conditions for the electrochemical reduction of carbon dioxide (CO2RR) are summarized in Figure 5 [18,22,23,24,25,26,27,28,29,30,31,32,33].

According to the literature, catalysts such as CdS, MoS_2_, and Ag_2_S show a lower overpotential to award a high faradaic efficiency for producing CO. We refer this to the crystal structure of the catalyst and the conductive properties.

### Strategies to Improve CO Production Efficiency

The efficiency of carbon monoxide (CO) production by electrochemical reduction of carbon dioxide (CO_2_) is influenced by several factors, including catalyst activity and selectivity, electrode morphology, and electrolyte composition. To improve the efficiency of CO production, researchers have developed strategies to optimize these parameters through catalyst engineering, electrode design, and electrolyte optimization. By adjusting the properties of catalysts, electrodes, and electrolytes, it is possible to improve CO selectivity, Faradaic efficiency, and overall process performance [15].

Catalyst engineering is about the development and synthesis of catalyst materials with tailored properties to optimize CO_2_ reduction kinetics and selectivity. Strategies such as doping, alloying, and nanostructuring can increase catalyst activity and stability and, thus, improve the efficiency of CO production. Similarly, electrode design plays a crucial role in optimizing mass transfer, charge transfer, and surface area, which are essential for efficient CO_2_ electroreduction. By controlling the electrode morphology, composition, and architecture, it is possible to increase CO selectivity and minimize competing side reactions [15].

Optimizing electrolyte composition and operating conditions is another key strategy to improve CO production efficiency. Electrolytes play a critical role in facilitating CO_2_ reduction reactions by providing ions for charge transfer and stabilizing reaction intermediates. By adjusting the pH, concentration and composition of the electrolyte, reaction kinetics and product selectivity can be modulated. In addition, the optimization of operating parameters such as temperature, pressure, and flow rate can further improve the efficiency of CO_2_ electroreduction [3]. The reason behind this is that temperature increases reaction rates by providing the necessary thermal energy, which accelerates CO_2_ conversion. However, it must be carefully regulated to avoid overheating and damage to the system’s components. Pressure affects the CO_2_ concentration at the electrode by increasing its partial pressure, which enhances the reaction rate. Higher pressure improves CO_2_ availability but requires robust equipment to manage the added mechanical stresses. Flow rate ensures a steady supply of CO_2_ to the electrode and efficient removal of reaction products. Proper flow management helps maintain a high reactant concentration and prevents product buildup that could inhibit the reaction.

## 5. Role of Membranes in CO_2_ Electrolyzers

In CO_2_ electrolyzers, membranes are integral to the system’s ability to convert CO_2_ into valuable chemicals and fuels. They serve as selective barriers that separate the anode and cathode compartments, allowing ion transport while preventing the undesired crossover of gasses like CO_2_, H_2_, or O_2_. This selectivity is essential for high Faradaic efficiency (i.e., the efficiency of charge transfer in producing desired products) and for minimizing side reactions that can degrade the electrolyzer’s performance over time [34,35,36].

Three common classes of membranes are used in a CO_2_ flow reactions. Based on the ionic functional groups attached to polymer chains, ion exchange membranes (IEM) are divided into anion exchange membranes (AEM), cation exchange membranes (CEM), and bipolar membranes (BPM). The type of membrane has an influence on the pH of both sides of the membrane. Based on the pH impact, the reactant availability and reaction potential of cathodic as well as anodic reactions changes. CEM is used to transport cations from an acidic anode to the cathode. In contrast to the CEM, AEM transport anions from the basic cathode to the anode. BPM leads to dissociation of H_2_O in order to enable the transportation of H^+^ to the cathode and the OH^-^ to the anode or transportation of H^+^ from the anode and OH^-^ from the cathode and the formation of water at the center of membrane [37].

### 5.1. Proton Exchange Membranes (PEM)

Proton exchange membranes are commonly used in acidic electrolysis environments. They facilitate the movement of protons (H^+^) from the anode to the cathode while preventing other species, such as CO_2_, from crossing. Nafion^®^ is a widely used PEM due to its high ionic conductivity and chemical stability under acidic conditions. However, environments in PEM cells can limit the choice of catalysts and present challenges for CO_2_ reduction due to possible catalyst degradation. PEMs also show CO_2_ crossover, which can affect reaction selectivity and reduce overall efficiency [38,39].

A cation exchange membrane is suitable for a zero-gap CO_2_ electrolyzer system. A big advantage is that carbonation can be avoided, and carbon efficiency is improved. Different cation exchange membranes are based on Nafion^®^ membranes (111, 112, 115, 211, and 212) with different thicknesses. Higher FECO, as well as energy efficiency, is achieved by thinner Nafion^®^ membranes. Non-acidic anolyte promotes the formation of carbonates and bicarbonates in the gas diffusion electrode, which would lead to clogging of the flow field channel as well as blocking of the catalytic surfaces. On the other hand, acidic anolyte remains an issue due to high hydrogen production [40].

#### 5.1.1. Structure and Application of CEMs in CO2RR

CEMs are designed to selectively transport cations, such as protons (H^+^), while blocking anions. They are commonly employed in batch-type H-cell reactors for initial catalyst development, where CO_2_-saturated aqueous electrolytes are used. CEMs enable efficient conversion of CO_2_ into valuable products such as carbon monoxide (CO), formic acid (HCOOH), methane (CH_4_), and alcohols under near-neutral to alkaline conditions. Nafion™, a widely used PFSA-based CEM, has shown remarkable Faradaic efficiencies (FE) for these reactions, with up to 98% for formic acid and 97% for CO. While flow reactors have begun adopting CEMs, their application is still less prevalent compared to batch-type setups [36,41,42].

In flow electrolyzers, CEMs inhibit anionic product crossover, making them particularly suitable for producing formate (HCOO−). Novel cell designs leveraging the high proton conductivity of CEMs have achieved significant reductions in ohmic losses. For instance, using Nafion™ in a zero-gap configuration allows for high CO partial current densities with remarkably low cell voltages. Moreover, hybrid cell structures with buffer layers between the CEM and cathode provide opportunities to suppress undesired hydrogen evolution reactions (HER), albeit at the cost of increased energy losses due to additional resistance [43,44,45].

#### 5.1.2. Proton Conductivity

The core functionality of CEMs lies in their ability to transport protons efficiently. Proton conductivity in these membranes is governed by mechanisms such as the Grotthuss mechanism, where protons “hop” between hydrolyzed anionic sites through the formation of hydronium ions. This mechanism is faster than vehicular transport, making it ideal for CEM-based systems. Factors influencing proton conductivity include the ion exchange capacity (IEC), water uptake, and the morphology of ionic channels. For example, perfluorosulfonic acid (PFSA)-based CEMs like Nafion™ exhibit high proton conductivities (>100 mS/cm) due to their phase-separated structure, which forms a highly interconnected network of hydrated ion channels [46].

Improving proton conductivity involves increasing the IEC, which enhances the density of ionic charges within the membrane. However, this must be balanced against potential drawbacks, such as excessive swelling and mechanical instability [36].

#### 5.1.3. Cation Crossover and CO2RR Selectivity

In CO2RR systems, cation crossover is a critical factor influencing product selectivity. The transport of cations such as K^+^, Na^+^, and Cs^+^ through CEMs can affect CO_2_ reduction kinetics by altering the local electric field and enhancing CO_2_ adsorption. Research has demonstrated that using CEMs saturated with specific alkali cations can improve selectivity for certain products, such as CO, by suppressing proton transport and reducing competing reactions like HER [46,47,48,49].

However, cation crossover also poses challenges. For example, the leaching of substituted cations to the cathode can impact long-term stability and necessitate post-electrolysis purification. Achieving a balance between optimizing cation transport for selectivity, and minimizing undesired crossover remains an area of active research [47,48,49,50].

#### 5.1.4. Product Crossover

Product crossover is a significant limitation in scaling CO2RR systems for industrial applications. Neutral products, such as alcohols, can diffuse through CEMs, contaminating the anolyte and increasing the complexity of downstream separation processes. Unlike anion exchange membranes (AEMs), CEMs exhibit electroosmotic drag (EOD) of water toward the cathode, mitigating anolyte contamination. However, this advantage is offset by challenges associated with high alcohol permeability and acidic product crossover [51,52,53,54,55,56].

Strategies to minimize product crossover include modifying PFSA membranes with inorganic materials, such as silica or zirconium phosphate, or employing nonfluorinated polymers with reduced permeability. Surface functionalization and polymer crosslinking are additional approaches to enhance selectivity and reduce crossover [36,57,58,59].

#### 5.1.5. Stability and Durability

The long-term stability of CEMs is crucial for their viability in commercial CO2RR systems. Membrane degradation, often caused by radical-induced chain scission or excessive swelling, leads to a decline in ionic conductivity and mechanical integrity. In PEM water electrolyzers, strategies such as incorporating radical scavengers or using hydrocarbon-based membranes have shown promise in mitigating degradation. These approaches can be adapted for CEMs in CO2RR systems.

Hydrocarbon-based CEMs offer lower gas crossover rates but are more susceptible to free radical attacks than PFSA membranes. Advances in polymer chemistry, such as the development of sulfonated polyphenylenes, aim to address this trade-off by improving both chemical stability and ionic conductivity. However, achieving high stability while maintaining performance remains a key challenge [60,61].

#### 5.1.6. Summary and Future Directions for CEM

CEMs have significant potential for enabling efficient and scalable CO_2_ electrolysis systems. Their high proton conductivity, low anionic crossover, and compatibility with various electrolyzer designs make them an attractive choice for CO2RR applications. However, challenges such as excessive swelling, neutral product crossover, and susceptibility to degradation under acidic conditions must be addressed [62,63].

Future research should focus on the following:

- Material Innovations: Developing novel CEM materials with tailored properties, such as improved ionic selectivity, reduced permeability, and enhanced durability;
- Cell Design Optimization: Integrating buffer layers or hybrid structures to suppress HER while minimizing energy losses;
- Scalability and Cost Reduction: Reducing the reliance on expensive PFSA-based membranes by exploring nonfluorinated alternatives and scalable fabrication methods;
- Stability Enhancements: Incorporating radical scavengers, surface modifications, and crosslinking to improve long-term performance.

In conclusion, while CEMs offer promising advantages for CO2RR, addressing their limitations through material science and engineering innovations will be critical for realizing their full potential in sustainable carbon utilization technologies [64,65].

### 5.2. Anion Exchange Membranes (AEM)

AEMs are typically employed in alkaline CO_2_ electrolyzers, where they allow hydroxide ions (OH^−^) to transport from the cathode to the anode. This alkaline environment has advantages for CO_2_ reduction reactions as it can improve reaction rates and enhance product selectivity, especially for multi-carbon products. However, AEMs face issues with stability in alkaline solutions, as they can degrade over time, particularly when exposed to high pH and carbonate ions that form when CO_2_ dissolves in the electrolyte. Efforts are underway to discover AEMs with improved chemical and mechanical stability by using novel polymer backbones and functional groups that resist degradation [36,66].

Membranes contain layers that conduct both H^+^ and OH^−^ ions, allowing them to create a locally acidic environment at the cathode and an alkaline environment at the anode. This dual environment can help balance the pH gradients across the cell, which is beneficial for sustaining high activity in both half reactions. BPMs offer flexibility in reaction tuning, leading to higher selectivity for products like formate and ethylene, depending on catalyst choice and operating conditions. However, these membranes also face challenges related to water dissociation and require optimization to minimize energy losses associated with ion transport [36,67].

In Table 3 the comparison of different membrane types are described. By comparing CEMs, AEMs, and BPMs, CEMs has high conductivity but an excessive swelling as well as low faradaic efficiency. AEMs has a high faradaic efficiency, but the main concern is the product and the carbonate ion crossover. The BPM indicates minimal crossover but has a higher resistance as well as delamination [36].

The recent status of the IEM-based CO2RR reactors show cation exchange membranes as being mostly used in batch-type reactors. This process is used to test new developed electrocatalysts. In order to test the design and tuning of selectivity, H-cells also utilize CEM. The recent trend is that the CEM is also used in flow reactors to investigate and overcome the challenges associated with CEMs covering flux of protons toward the cathode during a continuous electrolysis producing hydrogen over the CO2RR [36].

In order to overcome the challenges related to CEMs, AEMs are also investigated, especially when alkaline conditions are chosen. Therefore, different alkaline stable and conductive AEM materials are developed. AEMs are more popularly chosen than CEMs by usage in flow CO_2_ electrolyzers [36]. In Table 4 the state-of-the-art performance metrics of flow CO2RR electrolyzers are summarized.

A new approach is to use an IEM as a BPM. This membrane has a laminating cation exchange layer as well as an anion exchange layer. Therefore, cationic and anionic mobile counterions are transported through their respective segments either to form water or transport from the interface where a rapid dissociation of water happens. BPMs are applied in reactors using liquid bicarbonate as feedstock [36].

#### 5.2.1. Anion Exchange Membranes (AEMs)

Anion Exchange Membranes (AEMs) are key components in electrochemical systems such as fuel cells, electrolyzers, and CO_2_ reduction cells. Their efficiency and stability are critical in determining the overall performance of these systems, especially in applications that operate under high pH conditions. A thorough understanding of the behavior of AEMs in these environments reveals several important aspects, which include selectivity in CO_2_ reduction reactions (CO2RR), rapid carbonation reactions, ionic conductivity, product crossover, and system stability [36].

#### 5.2.2. CO2RR Selectivity Under High-pH Conditions

One of the central concerns in using AEMs for CO2RR is maintaining high selectivity for the desired products under high pH conditions. At an elevated pH, the solubility and the reactivity of CO_2_ changes significantly. AEMs, which are typically used to transport anions such as hydroxide ions (OH^−^), facilitate CO2RR by enabling the transport of bicarbonate (HCO_3_^−^) and carbonate (CO_3_^2^^−^) ions within the electrolyte. However, in high-pH environments, the increased presence of OH^−^ ions can compete with CO_2_, potentially leading to undesirable side reactions. Optimizing AEMs involves adjusting their composition and structure to reduce such competition, thereby increasing the efficiency and selectivity for CO2RR products such as carbon monoxide (CO), methane (CH_4_), or other carbon-based chemicals [77,78,79,80,81].

#### 5.2.3. Rapid Carbonation Reactions

AEMs are susceptible to carbonation reactions due to the affinity of the membrane material for CO_2_. These carbonation reactions can significantly affect the membrane’s performance by altering its ionic conductivity and mechanical integrity. Under high-pH conditions, where CO_2_ is readily available, the formation of bicarbonate and carbonate ions is enhanced. The reaction between CO_2_ and the membrane material often leads to the formation of ionic clusters that can increase resistance and reduce the overall ionic conductivity of the AEM. Thus, controlling the rate of carbonation is critical to maintain the efficiency of the electrochemical process. Strategies to mitigate carbonation include modifying the chemical structure of the membrane or employing additives that hinder CO_2_ absorption without compromising ionic conductivity [50,82].

#### 5.2.4. Ionic Conductivity

Ionic conductivity is a key performance indicator for AEMs, as it directly impacts the efficiency of the electrochemical reactions. AEMs rely on the mobility of anions (typically hydroxide or carbonate ions) for ion transport within the system. High ionic conductivity is crucial for minimizing ohmic losses and ensuring efficient electron transfer across the system. However, the ionic conductivity of AEMs can be significantly impacted by high-pH conditions due to the formation of insoluble carbonate species or by changes in membrane structure resulting from carbonation. The development of AEMs with enhanced ionic conductivity under such harsh conditions is a primary goal of ongoing research. This can be achieved by modifying the polymer backbone, incorporating highly conductive ionic groups, or introducing fillers that promote ion transport without introducing detrimental effects on the membrane’s mechanical properties [50,83,84,85].

#### 5.2.5. Product Crossover

In electrochemical systems, product crossover refers to the undesired transport of reaction products across the membrane from one side of the electrochemical cell to the other. In AEM-based systems, the main concern is the crossover of products such as CO, H_2_, or other gasses, which can lead to efficiency losses and degradation of the system. High-pH conditions can exacerbate this issue by increasing the solubility of certain products or by altering the properties of the AEM. To minimize product crossover, it is essential to design membranes with appropriate ion-exchange capacities, porosities, and surface chemistries that can selectively allow the passage of ions without permitting gasses or other products to diffuse through the membrane. Enhancing the selectivity of AEMs is critical for ensuring high product yields and maintaining the overall system’s stability and efficiency [86,87].

#### 5.2.6. System Stability

The long-term stability of AEMs under operating conditions is another important factor. High-pH environments, especially when combined with the presence of CO_2_, can degrade the membrane materials over time. This degradation can manifest as mechanical breakdown, reduced ionic conductivity, or increased susceptibility to carbonation. In CO2RR applications, maintaining membrane integrity over long periods is crucial for ensuring stable and reliable performance. Strategies to enhance system stability include optimizing the chemical composition of the membrane, utilizing protective coatings, or incorporating stabilizing agents that prevent the formation of harmful ionic species. Additionally, improving the robustness of the membrane under high-pH conditions helps to extend the lifespan of the electrochemical system and reduce maintenance costs [36,88].

#### 5.2.7. Summary and Future Directions for AEMs

In summary, AEMs play a critical role in electrochemical applications, especially in CO_2_ reduction reactions, by facilitating the transport of anions like hydroxide and carbonate. Their performance is influenced by a variety of factors, including the selectivity for desired products, the occurrence of carbonation reactions, their ionic conductivity, the potential for product crossover, and their overall stability under high-pH conditions. Addressing these challenges requires continuous advancements in membrane design, material science, and process optimization to improve selectivity, minimize side reactions, and enhance long-term stability. As research progresses, it is expected that AEMs will continue to evolve, offering more efficient and sustainable solutions for CO2RR and other electrochemical applications [36].

### 5.3. Bipolar Membranes (BPMs)

Bipolar membranes (BPMs) are an emerging class of ion exchange membranes (IEMs) used in electrochemical systems, particularly for CO_2_ reduction reactions (CO2RR). BPMs are composed of a cation exchange layer (CEL) and an anion exchange layer (AEL), which together form a unique structure capable of supporting different pH environments on either side of the membrane, crucial for CO2RR processes. This pH difference is established through the dissociation of water at the interface between the CEL and AEL, driven by an electric field. Depending on the direction of the applied voltage, BPMs can operate with reverse or forward bias, each mode having specific advantages and challenges for CO2RR [89,90,91,92].

Water Dissociation and Overpotentials: The reverse bias mode of BPMs facilitates water dissociation at the CEL-AEL interface, generating protons and hydroxide ions that are transported across the respective layers. This electrochemical process is essential for maintaining ionic conductivity, especially under conditions where water splitting is necessary, such as in CO2RR or water electrolysis. However, the overpotentials associated with water dissociation remain significant, particularly at current densities above 20 mA cm^−2^. For optimal performance, efficient water dissociation catalysts are needed at the interface to lower the overpotentials, as these can exceed 100 mV even at moderate current densities. Studies have shown that employing specific metal oxide or polymer-based catalysts at the CEL-AEL interface can improve the efficiency of water dissociation, enabling better performance under high current densities [93,94].

Anionic and Neutral Product Crossover: A significant advantage of BPMs in CO2RR reactors is their ability to limit the crossover of anionic species such as HCO_3_^−^ and CO_3_^2−^, which can negatively affect reactor performance. In reverse bias, the protonation of bicarbonate and carbonate species helps prevent their crossover to the anode, facilitating CO_2_ capture. This selective exclusion is essential in ensuring the efficiency of CO2RR, where the presence of unwanted ions can decrease the Faradaic efficiency (FE). However, under forward bias, neutral products and CO_2_ produced at the interface can lead to accumulation issues if not efficiently removed, causing potential membrane degradation [36,85,95,96].

Mechanical Degradation and Delamination: The mechanical stability of BPMs is a crucial consideration in their development, particularly under harsh electrochemical conditions. The different chemical and physical properties of the CEL and AEL can lead to mechanical degradation, such as delamination of the two layers. This can occur due to internal stressors like temperature fluctuations, pressure differentials, and water accumulation at the interface, particularly during forward bias operation. Delamination is further exacerbated by the formation of water and CO_2_ at the interface in the forward bias, which can cause structural breakdown if not properly managed. Research efforts have focused on improving the adhesion between the layers, using strategies such as electrospinning to enhance the interfacial area and prevent delamination. Additionally, the differential thickness of the layers has been explored to mitigate water transport limitations and reduce mechanical degradation [89].

Recent Developments and Applications: Recent studies have explored novel approaches to improve BPM performance. For instance, electrospinning has been used to create three-dimensional (3D) BPM structures that offer improved mechanical integrity and catalytic performance. The 3D structure increases the interfacial area, which is crucial for effective water dissociation and for preventing localized dehydration. These advanced BPMs are designed to accommodate high-current density operations, which are necessary for industrial-scale CO2RR reactors. Moreover, modifications to the membrane structure, such as roughening the interfaces between the CEL and AEL, have shown promise in reducing mechanical failures and enhancing performance under high-stress conditions [36].

Challenges and Future Directions: Despite the potential of BPMs, several challenges remain in their application for CO2RR. The high cell voltages required in reverse bias configurations, especially under conditions of low pH at the cathode, limit their efficiency. Additionally, the low Faradaic efficiencies observed in CO2RR reactors using BPMs, due to parasitic hydrogen evolution reactions (HER) at the cathode, must be addressed. However, the use of liquid CO_2_ feedstocks and strategies to improve the local pH environment at the cathode can help mitigate some of these challenges. Further development of water dissociation catalysts, membrane design improvements, and reactor configurations will be necessary to make BPMs a viable option for CO2RR at industrial scales [36].

#### Summary of the Ion Exchange Membranes

BPMs represent a promising alternative to traditional anion exchange membranes (AEMs) and cation exchange membranes (CEMs) for CO2RR applications. Their ability to control pH at both the anode and cathode, minimize product crossover, and provide a mechanism for water dissociation at the CEL-AEL interface makes them particularly suitable for CO2RR systems. However, challenges related to overpotentials, mechanical degradation, and the need for efficient water dissociation catalysts remain significant hurdles to their commercial application. Future research focusing on improving BPM performance, including the development of more robust materials, better catalysts, and optimized reactor designs, will be essential to realizing their full potential in CO_2_ reduction and other electrochemical applications.

### 5.4. Performance and Challenges

The performance of electrolyzers depends heavily on membrane characteristics such as ionic conductivity, selectivity, chemical stability, and gas separation properties [36].

Ion Conductivity: High conductivity reduces resistive losses, allowing more efficient transport of ions without needing high operating voltages, which can otherwise lead to undesirable side reactions [36].

Chemical Stability: The acidic and alkaline environments in PEM and AEM electrolyzers, respectively, present harsh conditions that can degrade membrane materials over time. Improvements in stability are crucial for reducing maintenance costs and enhancing long-term durability [36,97].

Gas Crossover: Preventing the crossover of CO_2_ and H_2_ for maintaining product purity and minimizing side reactions. Developing membranes with low gas permeability but high ionic conductivity is an ongoing research focus [36,98].

Membrane degradation from high current densities and carbonate buildup (especially in alkaline environments with AEMs and BPMs) can affect system stability. For example, carbonate formation in AEMs reduces CO_2_ availability for the reduction reaction, leading to lower overall efficiency [36].

## 6. Challenges and Future Directions

Identifying the key challenges hindering the widespread adoption of electrochemical CO_2_ reduction technologies is important.

Despite the considerable progress that has been made in electrochemical carbon dioxide (CO_2_) reduction, there are a number of challenges that hinder the adoption of this technology for CO production on an industrial scale. These challenges include technical, economic, and regulatory barriers that need to be addressed in order to realize the full potential of electrochemical CO_2_ reduction as a sustainable route to CO production. Regulatory barriers that hinder the adoption of electrochemical CO_2_ reduction technology include the lack of specific financial incentives, inconsistent regulations across regions, and stringent environmental standards. Additionally, complex carbon emission reporting requirements, restrictions on CO_2_ storage, lengthy approval processes, and intellectual property issues further complicate the technology’s widespread implementation. These obstacles collectively impact the technology’s feasibility and delay its industrial adoption.

Key research challenges include the development of efficient and selective catalysts, optimization of reactor design and operation, integration with renewable energy sources, and scalability of CO_2_ electroreduction processes.

One of the biggest challenges in CO_2_ electroreduction is the development of catalysts that exhibit high activity, selectivity, and stability under realistic operating conditions. Despite advances in catalyst synthesis and characterization, precise control of reaction pathways and product distributions remain a challenging task. In addition, the integration of CO_2_ electroreduction with renewable energy sources such as solar and wind energy poses logistical and technical challenges related to intermittency, grid integration, and energy storage.

Economic considerations also play an important role in the commercial viability of CO_2_ electroreduction technologies. The high capital costs associated with electrochemical reactors, catalyst materials, and infrastructure pose financial barriers to widespread adoption. In addition, uncertainties related to market demand, regulatory frameworks, and political incentives complicate investment decisions and technology deployment strategies.

The economic assessment of electrochemical CO_2_ reduction methods in comparison with non-electrochemical CO production technologies is a crucial area of study. Such analyses provide valuable insights into the cost competitiveness and scalability of these emerging technologies. By evaluating the potential economic advantages of electrochemical methods, researchers can better understand their viability and the factors driving their adoption. Moving forward, it is essential to prioritize this aspect to comprehensively highlight the practical implications and transformative potential of electrochemical CO_2_ reduction in addressing global CO production demands.

## 7. Conclusions

In summary, the electrochemical reduction of carbon dioxide (CO_2_) represents a promising pathway for sustainable carbon monoxide (CO) production with implications for climate change mitigation, industrial decarbonization, and renewable energy integration. By utilizing renewable electricity and captured CO_2_ emissions, electrochemical CO_2_ reduction enables the synthesis of CO with high efficiency and selectivity, reducing dependence on fossil fuels and contributing to a circular carbon economy.

In this review, we have examined the mechanisms, catalysts, strategies, and applications of electrochemical CO_2_ reduction, highlighting its potential to address pressing environmental issues while creating economic value. A particular focus was placed on the critical role of ion exchange membranes (IEMs), including cation exchange membranes (CEMs), anion exchange membranes (AEMs), and bipolar membranes (BPMs). We discussed their advantages, such as improved ion transport and system stability, as well as their drawbacks, including material degradation and ion crossover. The current challenges associated with integrating these membranes into CO_2_ electroreduction systems, such as cost, scalability, and long-term performance, were also highlighted.

In the future, continued investment in research, development, and commercialization is essential to overcome the remaining challenges and realize the full potential of electrochemical CO_2_ reduction technologies. This includes advancing the design, functionality, and durability of ion exchange membranes, which are key to optimizing reactor performance and CO production efficiency. By fostering collaboration between academia, industry, and government, it is possible to accelerate innovation, scale up production, and create new markets for CO-derived products. Ultimately, the transition towards sustainable CO production requires concerted efforts from all stakeholders to build a resilient, resource-efficient, and low-carbon economy for future generations.

## Figures and Tables

**Table 1:** Comparison of electrochemical and photochemical CO_2_ reduction technologies.

Aspect	Electrochemical CO_2_ Reduction	Photochemical CO_2_ Reduction
Advantages		
Selectivity and Efficiency	High selectivity towards specific products like CO or ethylene, especially with advanced catalysts (e.g., copper-based) [9,10].	Utilizes solar energy directly, offering a potentially more sustainable approach if efficient catalysts and systems are developed [9].
Scalability	Easier to scale and integrate with renewable energy sources such as solar and wind, making it suitable for large-scale applications [10].	Potential for simpler system design, particularly in regions with abundant sunlight [9].
Energy Input Control	Precise control over applied voltage allows optimization of the reduction process and management of energy input, enhancing efficiency [9].	Direct harnessing of sunlight can make the process more cost-effective and sustainable if material and system efficiency is improved [9].
Disadvantages		
Energy Demand	High energy requirements, especially for producing multi-carbon products, which necessitate significant energy input [10].	Lower efficiency and selectivity compared to electrochemical methods, limiting the production of high-value products at scale [9].
System Complexity	Requires complex system designs with precise control over reaction conditions, adding to the operational complexity and cost [9].	Highly dependent on sunlight availability and intensity, making it less reliable in regions with variable sunlight exposure [9].
Material Degradation	Electrodes can degrade over time in harsh conditions, leading to increased costs and reduced system efficiency [9].	Photocatalysts may degrade under prolonged sunlight exposure, reducing system lifespan and increasing maintenance needs [9].
Environmental Impact	Depending on the energy source, the process may have a high environmental impact if non-renewable electricity is used [9].	Potentially lower environmental impact if stable and efficient photocatalysts that are environmentally benign are developed and utilized [9].

**Table 2:** Summary of Cu-based catalysts [20].

Catalysts	Products	Potential/ V vs. RHE	Current Density/ mA cm^−2^	FE/%	Electrolyte
Cu-NPs	CH_4_	−1.35	~9	76	0.1 M NaHCO_3_
CuPc	C_2_H_4_	−1.38	2.8	25	0.5 M KCl
CuDAT-wire samples	C_2_H_4_ C_2_H_5_OH	−0.8	90	40% C_2_H_4_, 20% C_2_H_5_OH	1 M KHCO_3_
Cu-N-substituted arylpyridinium	C_2_H_4_ C_2_H_5_OH	−1.1	1.02	78.2 (C > 1)	0.1 M KHCO_3_ + 10 mM additive
Cu(II)-NU-1000	Formate CO	−0.8 to −1.0	1.2	30	0.1 M NaClO_4_
Cu/SnO_2_	CO	−0.7	4.6	93	0.5 M KHCO_3_
AuCu alloy nanoparticles	CO	−0.77	1.39	80	0.1 M KHCO_3_
Cu dendrite	C_2_H_4_, C_2_H_6_	−0.8	5–7	55	1 M Na_2_SO_4_
N-C/Cu	C_2+_	−0.86	300	93	1 M KOH
Oxygen-bearing Cu	C_2_H_4_	−1.0	44.7	45	0.5 M KHCO_3_
Sn-Cu/SnO_x_	Formate and CO	−0.7	243	98	1 M KOH
Cu_2_O@CuHHTP	CH_4_	−1.4	11	73	0.1 M KCl 0.1 M KHCO_3_
Cu_2_S	HCOOH	−0.9	19	87	0.1 M NaHCO_3_
Cu_2_O/Cu_2_S	HCOOH	−0.9	15.3	67.6	0.1 M KHCO_3_

**Table 3:** Comparision of different membrane types including properties [34,35,36,37,38,39,40].

Membrane Type	Transported Ion	Operating pH	Conductivity	Thickness	Advantages	Challenges
PEM	H^+^	Acidic	50–100 mS/cm at 80 °C	20–50 µm	High ionic conductivity; effective in suppressing side reactions; suitable for compact designs.	Limited stability in acidic conditions; catalyst options limited by acidic environment; CO_2_ crossover.
AEM	OH^−^	Alkaline	20–80 mS/cm at room temperature	25–100 µm	Favorable for CO_2_ reduction rates and multi-carbon product selectivity; reduced catalyst degradation in alkaline environment.	Vulnerable to carbonate buildup; long-term stability issues in high-pH environments.
BPM	H^+^ and OH^−^	Acidic/Alkaline (bipolar)	5–20 mS/cm per layer (H^+^ and OH^−^ layers)	50–200 µm (combined thickness)	Allows local pH tuning (acidic at one electrode, alkaline at the other); improved selectivity for target products.	Water dissociation increases energy loss; sensitivity to ion imbalance and membrane swelling.

**Table 4:** State-of-the-art performance metrics of flow CO2RR electrolyzers using different types of IEMs.

IEM	IEM Type	Cathode Feed	Anode Feed	Base Metal of CO_2_RR Catalyst	*E*_cell_/V	Main Product (FE/%)	Total Current Density /(mA cm^−2^)	Stability	Ref.
Nafion™ 117	CEM	CO_2_ + 2 M KHCO_3_	2 M KOH	Ag	3.9–4.9	CO (90)	220	10 h, r. t. ^b^(room temperature)	[68]
Nafion^TM^ 117	CEM	CO_2_ + 2 M KCl	2 M KOH	Cu	3.30	C_2+_ (80)	150	30 h, r. t.	[69]
Nafion^TM^ 212	CEM	Dry CO_2_	Humidified H_2_	Sn	2.47	HCOOH (72)	385	1 h, r. t.	[69]
PiperION TP-85	AEM	Humidified CO_2_	0.1 M CsOH	Ag	3.20	CO (85)	>1000	100 h (60 °C)	[70]
Aemion^TM^ AF1-CNN8-60-X	AEM	Humidified CO_2_	0.1 M KHCO_3_	Cu	3.75	CH_3_OH + C_3_H_7_OH (–)	150	100 h (40 °C)	[71]
Sustainion^®^ X37-50	AEM	Humidified CO_2_	0.01 M KHCO_3_	Ag	2.89	CO (99)	100	>3000 h (hours)	[72]
Selemion^TM^ DSVN	AEM	CO_2_ + 1 M KHCO_3_	1 M KHCO_3_	Ni	–	CO (98)	400	30 h	[73]
QAPPT	AEM	CO_2_ + 10 mM CsOH	10 mM CsOH	Ag	3.3	CO (90)	550	100 h	[74]
Fumasep^®^ FBM	BPM	3 M KHCO_3_	1 M KOH	Ag	3.5	CO (82)	100	8 h, r. t.	[75]
Fumasep^®^ FBM-PK	BPM	CO_2_ (40 bar) + 0.5 M KHCO_3_	1 M KOH	Sn	3.5	HCOOH (90)	30	1/3 h, r. t.	[76]

## References

[1] Treibhausgase. Zugegriffen: 19. https://www.umweltbundesamt.at/klima/treibhausgase.

[2] Detz R.J., Ferchaud C.J., Kalkman A.J., Kemper J., Sánchez-Martínez C., Saric M., Shinde M.V. (2023). Electrochemical CO_2_ conversion technologies: State-of-the-art and future perspectives. Sustain. Energy Fuels.

[3] Liu Z., Qian J., Zhang G., Zhang B., He Y. (2024). Electrochemical CO_2_-to-CO conversion: A comprehensive review of recent developments and emerging trends. Sep. Purif. Technol..

[4] Nitopi S., Bertheussen E., Scott S.B., Liu X., Engstfeld A.K., Horch S., Seger B., Stephens I.E.L., Chan K., Hahn C. (2019). Progress and Perspectives of Electrochemical CO_2_ Reduction on Copper in Aqueous Electrolyte. Chem. Rev..

[5] Draxler M., Schenk J., Bürgler T., Sormann A. (2020). The Steel Industry in the European Union on the Crossroad to Carbon Lean Production—Status, Initiatives and Challenges. BHM Berg-Hüttenmänn. Monatshefte.

[6] Bierhals J. (2001). Carbon Monoxide. Ullmann’s Encyclopedia of Industrial Chemistry.

[7] Liu Z., Yang H., Kutz R., Masel R.I. (2018). CO_2_ Electrolysis to CO and O_2_ at High Selectivity, Stability and Efficiency Using Sustainion Membranes. J. Electrochem. Soc..

[8] Küngas R. (2020). Review—Electrochemical CO_2_ Reduction for CO Production: Comparison of Low- and High-Temperature Electrolysis Technologies. J. Electrochem. Soc..

[9] Han G.H., Bang J., Park G., Choe S., Jang Y.J., Jang H.W., Kim S.Y., Ahn S.H. (2023). Recent Advances in Electrochemical, Photochemical, and Photoelectrochemical Reduction of CO_2_ to C^2+^ Products. Small.

[10] Wyndorps J., Ostovari H., von der Assen N. (2021). Is electrochemical CO_2_ reduction the future technology for power-to-chemicals? An environmental comparison with H_2_-based pathways. Sustain. Energy Fuels.

[11] Li X., Yu J., Jaroniec M., Chen X. (2019). Cocatalysts for Selective Photoreduction of CO_2_ into Solar Fuels. Chem. Rev..

[12] Lin J., Yan S., Zhang C., Hu Q., Cheng Z. (2022). Electroreduction of CO_2_ toward High Current Density. Processes.

[13] Lu S., Shi Y., Meng N., Lu S., Yu Y., Zhang B. (2020). Electrosynthesis of Syngas via the Co-Reduction of CO_2_ and H_2_O. Cell Rep. Phys. Sci..

[14] Liu C., Cundari T.R., Wilson A.K. (2012). CO_2_ Reduction on Transition Metal (Fe, Co, Ni, and Cu) Surfaces: In Comparison with Homogeneous Catalysis. J. Phys. Chem. C.

[15] Sun D., Xu X., Qin Y., Jiang S.P., Shao Z. (2020). Rational Design of Ag-Based Catalysts for the Electrochemical CO_2_ Reduction to CO: A Review. ChemSusChem.

[16] Hori Y., Murata A., Takahashi R. (1989). Formation of hydrocarbons in the electrochemical reduction of carbon dioxide at a copper electrode in aqueous solution. J. Chem. Soc. Faraday Trans. 1 Phys. Chem. Condens. Phases.

[17] Manthiram K., Beberwyck B.J., Alivisatos A.P. (2014). Enhanced Electrochemical Methanation of Carbon Dioxide with a Dispersible Nanoscale Copper Catalyst. J. Am. Chem. Soc..

[18] Verdaguer-Casadevall A., Li C.W., Johansson T.P., Scott S.B., McKeown J.T., Kumar M., Stephens I.E.L., Kanan M.W., Chorkendorff I. (2015). Probing the Active Surface Sites for CO Reduction on Oxide-Derived Copper Electrocatalysts. J. Am. Chem. Soc..

[19] Reske R., Mistry H., Behafarid F., Cuenya B.R., Strasser P. (2014). Particle Size Effects in the Catalytic Electroreduction of CO_2_ on Cu Nanoparticles. J. Am. Chem. Soc..

[20] Pei Y., Zhong H., Jin F. (2021). A brief review of electrocatalytic reduction of CO_2_—Materials, reaction conditions, and devices. Energy Sci. Eng..

[21] Zeng L., Shi J., Luo J., Chen H. (2018). Silver sulfide anchored on reduced graphene oxide as a high -performance catalyst for CO_2_ electroreduction. J. Power Sources.

[22] Liu S., Tao H., Liu Q., Xu Z., Liu Q., Luo J.-L. (2018). Rational Design of Silver Sulfide Nanowires for Efficient CO_2_ Electroreduction in Ionic Liquid. ACS Catal..

[23] He R., Zhang A., Ding Y., Kong T., Xiao Q., Li H., Liu Y., Zeng J. (2018). Achieving the Widest Range of Syngas Proportions at High Current Density over Cadmium Sulfoselenide Nanorods in CO_2_ Electroreduction. Adv. Mater..

[24] Li Y.H., Cheng L., Liu P.F., Zhang L., Zu M.Y., Wang C.W., Jin Y.H., Cao X.M., Yang H.G., Li C. (2018). Simple Cadmium Sulfide Compound with Stable 95% Selectivity for Carbon Dioxide Electroreduction in Aqueous Medium. ChemSusChem.

[25] Qin B., Li Y., Wang H., Yang G., Cao Y., Yu H., Zhang Q., Liang H., Peng F. (2019). Efficient electrochemical reduction of CO_2_ into CO promoted by sulfur vacancies. Nano Energy.

[26] Cheng L., Li Y., Chen A., Zhu Y., Li C. (2020). Impacts on carbon dioxide electroreduction of cadmium sulfides via continuous surface sulfur vacancy engineering. Chem. Commun..

[27] Xu J., Li X., Liu W., Sun Y., Ju Z., Yao T., Wang C., Ju H., Zhu J., Wei S. (2017). Carbon Dioxide Electroreduction into Syngas Boosted by a Partially Delocalized Charge in Molybdenum Sulfide Selenide Alloy Monolayers. Angew. Chem. Int. Ed. Engl..

[28] Huang W., Zhou D., Yang H., Liu X., Luo J. (2021). Dual-Doping Promotes the Carbon Dioxide Electroreduction Activity of MoS_2_ Nanosheet Array. ACS Appl. Energy Mater..

[29] Asadi M., Kumar B., Behranginia A., Rosen B.A., Baskin A., Repnin N., Pisasale D., Phillips P., Zhu W., Haasch R. (2014). Robust carbon dioxide reduction on molybdenum disulphide edges. Nat. Commun..

[30] Li C., Shen G., Zhang R., Wu D., Zou C., Ling T., Liu H., Dong C., Du X.-W. (2019). Zn nanosheets coated with a ZnS subnanometer layer for effective and durable CO_2_ reduction. J. Mater. Chem. A.

[31] Tetzlaff D., Pellumbi K., Puring K.J., Siegmund D., Polet W.S.K., Checinski M.P., Apfel U. (2021). Influence of the Fe: Ni Ratio in Fe_x_Ni_9-x_S_8_ (x = 3–6) on the CO_2_ Electroreduction. ChemElectroChem.

[32] Simon C., Zander J., Kottakkat T., Weiss M., Timm J., Roth C., Marschall R. (2021). Fast Microwave Synthesis of Phase-Pure Ni_2_FeS_4_ Thiospinel Nanosheets for Application in Electrochemical CO_2_ Reduction. ACS Appl. Energy Mater..

[33] Piontek S., Puring K.J., Siegmund D., Smialkowski M., Sinev I., Tetzlaff D., Cuenya B.R., Apfel U.-P. (2019). Bio-inspired design: Bulk iron–nickel sulfide allows for efficient solvent-dependent CO_2_ reduction. Chem. Sci..

[34] Gatto I., Patti A., Carbone A. (2023). Assessment of the FAA3-50 Polymer Electrolyte for Anion Exchange Membrane Fuel Cells. ChemElectroChem.

[35] Hasa B., Cherniack L., Xia R., Tian D., Ko B.H., Overa S., Dimitrakellis P., Bae C., Jiao F. (2023). Benchmarking anion-exchange membranes for electrocatalytic carbon monoxide reduction. Chem Catal..

[36] Habibzadeh F., Mardle P., Zhao N., Riley H.D., Salvatore D.A., Berlinguette C.P., Holdcroft S., Shi Z. (2023). Ion Exchange Membranes in Electrochemical CO_2_ Reduction Processes. Electrochem. Energy Rev..

[37] Salvatore D.A., Gabardo C.M., Reyes A., O’brien C.P., Holdcroft S., Pintauro P., Bahar B., Hickner M., Bae C., Sinton D. (2021). Designing anion exchange membranes for CO_2_ electrolysers. Nat. Energy.

[38] Fang W., Guo W., Lu R., Yan Y., Liu X., Wu D., Li F.M., Zhou Y., He C., Xia C. (2024). Durable CO_2_ conversion in the proton-exchange membrane system. Nature.

[39] ELees E.W., Mowbray B.A.W., Parlane F.G.L., Berlinguette C.P. (2022). Gas diffusion electrodes and membranes for CO_2_ reduction electrolysers. Nat. Rev. Mater..

[40] Sakita A.M., Ticianelli E.A. (2025). The role of cation exchange membrane characteristics in CO_2_ electrolysis to CO using acid anolyte. Electrochim. Acta.

[41] Ma W., Xie S., Liu T., Fan Q., Ye J., Sun F., Jiang Z., Zhang Q., Cheng J., Wang Y. (2020). Electrocatalytic reduction of CO_2_ to ethylene and ethanol through hydrogen-assisted C–C coupling over fluorine-modified copper. Nat. Catal..

[42] Sato M., Ogihara H., Yamanaka I. (2019). Electrocatalytic Reduction of CO_2_ to CO and CH4 by Co–N–C Catalyst and Ni co-catalyst with PEM Reactor. ISIJ Int..

[43] Vennekoetter J.-B., Sengpiel R., Wessling M. (2019). Beyond the catalyst: How electrode and reactor design determine the product spectrum during electrochemical CO_2_ reduction. Chem. Eng. J..

[44] Puring K.J., Evers O., Prokein M., Siegmund D., Scholten F., Mölders N., Renner M., Cuenya B.R., Petermann M., Weidner E. (2020). Assessing the Influence of Supercritical Carbon Dioxide on the Electrochemical Reduction to Formic Acid Using Carbon-Supported Copper Catalysts. ACS Catal..

[45] Rasul S., Pugnant A., Xiang H., Fontmorin J.-M., Yu E.H. (2019). Low cost and efficient alloy electrocatalysts for CO_2_ reduction to formate. J. CO2 Util..

[46] Luo T., Abdu S., Wessling M. (2018). Selectivity of ion exchange membranes: A review. J. Membr. Sci..

[47] Ersöz M. (1995). Diffusion and Selective Transport of Alkali Cations on Cation-Exchange Membrane. Sep. Sci. Technol..

[48] Resasco J., Chen L.D., Clark E., Tsai C., Hahn C., Jaramillo T.F., Chan K., Bell A.T. (2017). Promoter Effects of Alkali Metal Cations on the Electrochemical Reduction of Carbon Dioxide. J. Am. Chem. Soc..

[49] Ringe S., Clark E.L., Resasco J., Walton A., Seger B., Bell A.T., Chan K. (2019). Understanding cation effects in electrochemical CO_2_ reduction. Energy Environ. Sci..

[50] Huang J.E., Li F., Ozden A., Rasouli A.S., de Arquer F.P.G., Liu S., Zhang S., Luo M., Wang X., Lum Y. (2021). CO_2_ electrolysis to multicarbon products in strong acid. Science.

[51] Neburchilov V., Martin J., Wang H., Zhang J. (2007). A review of polymer electrolyte membranes for direct methanol fuel cells. J. Power Sources.

[52] Heinzel A., Barragán V.M. (1999). A review of the state-of-the-art of the methanol crossover in direct methanol fuel cells. J. Power Sources.

[53] Awang N., Ismail A., Jaafar J., Matsuura T., Junoh H., Othman M., Rahman M. (2015). Functionalization of polymeric materials as a high performance membrane for direct methanol fuel cell: A review. React. Funct. Polym..

[54] Ahmad H., Kamarudin S., Hasran U., Daud W. (2010). Overview of hybrid membranes for direct-methanol fuel-cell applications. Int. J. Hydrogen Energy.

[55] DeLuca N.W., Elabd Y.A. (2006). Polymer electrolyte membranes for the direct methanol fuel cell: A review. J. Polym. Sci. Part B Polym. Phys..

[56] Tschinder T., Schaffer T., Fraser S.D., Hacker V. (2007). Electro-osmotic drag of methanol in proton exchange membranes. J. Appl. Electrochem..

[57] Antonucci P.L., Aricò A.S., Cretì P., Ramunni E., Antonucci V. (1999). Investigation of a direct methanol fuel cell based on a composite Nafion^®^-silica electrolyte for high temperature operation. Solid State Ion..

[58] Vaivars G., Maxakato N.W., Mokrani T., Petrik L., Klavins J., Gericke G., Linkov V. (2004). Zirconium Phosphate Based Inorganic Direct Methanol Fuel Cell. Mater. Sci..

[59] Rao A.S., Rashmi K., Manjunatha D., Jayarama A., Shastrimath V.V.D., Pinto R. (2021). Methanol crossover reduction and power enhancement of methanol fuel cells with polyvinyl alcohol coated Nafion membranes. Mater. Today Proc..

[60] Adamski M., Peressin N., Holdcroft S. (2021). On the evolution of sulfonated polyphenylenes as proton exchange membranes for fuel cells. Mater. Adv..

[61] Klose C., Saatkamp T., Münchinger A., Bohn L., Titvinidze G., Breitwieser M., Kreuer K., Vierrath S. (2020). All-Hydrocarbon MEA for PEM Water Electrolysis Combining Low Hydrogen Crossover and High Efficiency. Adv. Energy Mater..

[62] Burton N., Padilla R., Rose A., Habibullah H. (2021). Increasing the efficiency of hydrogen production from solar powered water electrolysis. Renew. Sustain. Energy Rev..

[63] Masel R.I., Liu Z., Yang H., Kaczur J.J., Carrillo D., Ren S., Salvatore D., Berlinguette C.P. (2021). An industrial perspective on catalysts for low-temperature CO_2_ electrolysis. Nat. Nanotechnol..

[64] Besha A.T., Tsehaye M.T., Aili D., Zhang W., Tufa R.A. (2020). Design of Monovalent Ion Selective Membranes for Reducing the Impacts of Multivalent Ions in Reverse Electrodialysis. Membranes.

[65] Safronova E.Y., Golubenko D., Shevlyakova N., D’yakova M., Tverskoi V., Dammak L., Grande D., Yaroslavtsev A. (2016). New cation-exchange membranes based on cross-linked sulfonated polystyrene and polyethylene for power generation systems. J. Membr. Sci..

[66] Xu Q., Xu A., Garg S., Moss A.B., Chorkendorff I., Bligaard T., Seger B. (2023). Enriching Surface-Accessible CO_2_ in the Zero-Gap Anion-Exchange-Membrane-Based CO_2_ Electrolyzer. Angew. Chem. Int. Ed. Engl..

[67] De Mot B., Hereijgers J., Daems N., Breugelmans T. (2022). Insight in the behavior of bipolar membrane equipped carbon dioxide electrolyzers at low electrolyte flowrates. Chem. Eng. J..

[68] Duarte M., De Mot B., Hereijgers J., Breugelmans T. (2019). Electrochemical Reduction of CO_2_: Effect of Convective CO_2_ Supply in Gas Diffusion Electrodes. ChemElectroChem.

[69] Sen S., Brown S.M., Leonard M., Brushett F.R. (2019). Electroreduction of carbon dioxide to formate at high current densities using tin and tin oxide gas diffusion electrodes. J. Appl. Electrochem..

[70] Endrődi B., Kecsenovity E., Samu A., Halmágyi T., Rojas-Carbonell S., Wang L., Yan Y., Janáky C. (2020). High carbonate ion conductance of a robust PiperION membrane allows industrial current density and conversion in a zero-gap carbon dioxide electrolyzer cell. Energy Environ. Sci..

[71] Gabardo C.M., O’Brien C.P., Edwards J.P., McCallum C., Xu Y., Dinh C.-T., Li J., Sargent E.H., Sinton D. (2019). Continuous Carbon Dioxide Electroreduction to Concentrated Multi-carbon Products Using a Membrane Electrode Assembly. Joule.

[72] JKaczur J.J., Yang H., Liu Z., Sajjad S.D., Masel R.I. (2018). Carbon Dioxide and Water Electrolysis Using New Alkaline Stable Anion Membranes. Front. Chem..

[73] Chen K., Cao M., Lin Y., Fu J., Liao H., Zhou Y., Li H., Qiu X., Hu J., Zheng X. (2022). Ligand Engineering in Nickel Phthalocyanine to Boost the Electrocatalytic Reduction of CO_2_. Adv. Funct. Mater..

[74] Ye K., Zhang G., Ma X.-Y., Deng C., Huang X., Yuan C., Meng G., Cai W.-B., Jiang K. (2022). Resolving local reaction environment toward an optimized CO_2_-to-CO conversion performance. Energy Environ. Sci..

[75] Lees E.W., Goldman M., Fink A.G., Dvorak D.J., Salvatore D.A., Zhang Z., Loo N.W.X., Berlinguette C.P. (2020). Electrodes Designed for Converting Bicarbonate into CO. ACS Energy Lett..

[76] Zhou Y., Zhou R., Zhu X., Han N., Song B., Liu T., Hu G., Li Y., Lu J., Li Y. (2020). Mesoporous PdAg Nanospheres for Stable Electrochemical CO_2_ Reduction to Formate. Adv. Mater..

[77] Durst J., Siebel A., Simon C., Hasché F., Herranz J., Gasteiger H.A. (2014). New insights into the electrochemical hydrogen oxidation and evolution reaction mechanism. Energy Environ. Sci..

[78] Cheng T., Wang L., Merinov B.V., Goddard W.A. (2018). Explanation of Dramatic pH-Dependence of Hydrogen Binding on Noble Metal Electrode: Greatly Weakened Water Adsorption at High pH. J. Am. Chem. Soc..

[79] YHori Y., Takahashi R., Yoshinami Y., Murata A. (1997). Electrochemical Reduction of CO at a Copper Electrode. J. Phys. Chem. B.

[80] Liu X., Schlexer P., Xiao J., Ji Y., Wang L., Sandberg R.B., Tang M., Brown K.S., Peng H., Ringe S. (2019). pH effects on the electrochemical reduction of CO(_2_) towards C_2_ products on stepped copper. Nat. Commun..

[81] Kim C., Bui J.C., Luo X., Cooper J.K., Kusoglu A., Weber A.Z., Bell A.T. (2021). Tailored catalyst microenvironments for CO_2_ electroreduction to multicarbon products on copper using bilayer ionomer coatings. Nat. Energy.

[82] Keith D.W., Holmes G., St. Angelo D., Heidel K. (2018). A Process for Capturing CO_2_ from the Atmosphere. Joule.

[83] Ma M., Clark E.L., Therkildsen K.T., Dalsgaard S., Chorkendorff I., Seger B. (2020). Insights into the carbon balance for CO_2_ electroreduction on Cu using gas diffusion electrode reactor designs. Energy Environ. Sci..

[84] Unlu M., Zhou J., Kohl P.A. (2009). Anion Exchange Membrane Fuel Cells: Experimental Comparison of Hydroxide and Carbonate Conductive Ions. Solid-State Lett..

[85] Pătru A., Binninger T., Pribyl B., Schmidt T.J. (2019). Design Principles of Bipolar Electrochemical Co-Electrolysis Cells for Efficient Reduction of Carbon Dioxide from Gas Phase at Low Temperature. J. Electrochem. Soc..

[86] Li H., Oloman C. (2007). Development of a continuous reactor for the electro-reduction of carbon dioxide to formate—Part 2: Scale-up. J. Appl. Electrochem..

[87] Yang H., Kaczur J.J., Sajjad S.D., Masel R.I. (2017). CO_2_ Conversion to Formic Acid in a Three Compartment Cell with Sustainion^TM^ Membranes. ECS Trans..

[88] Laconti A., Liu H., Mittelsteadt C., McDonald R. (2006). Polymer Electrolyte Membrane Degradation Mechanisms in Fuel Cells—Findings Over the Past 30 Years and Comparison with Electrolyzers. ECS Trans..

[89] Pärnamäe R., Mareev S., Nikonenko V., Melnikov S., Sheldeshov N., Zabolotskii V., Hamelers H.V.M., Tedesco M. (2021). Bipolar membranes: A review on principles, latest developments, and applications. J. Membr. Sci..

[90] Oener S.Z., Foster M.J., Boettcher S.W. (2020). Accelerating water dissociation in bipolar membranes and for electrocatalysis. Science.

[91] Hohenadel A., Powers D., Wycisk R., Adamski M., Pintauro P., Holdcroft S. (2019). Electrochemical Characterization of Hydrocarbon Bipolar Membranes with Varying Junction Morphology. ACS Appl. Energy Mater..

[92] O’brien C.P., Miao R.K., Liu S., Xu Y., Lee G., Robb A., Huang J.E., Xie K., Bertens K., Gabardo C.M. (2021). Single Pass CO_2_ Conversion Exceeding 85% in the Electrosynthesis of Multicarbon Products via Local CO_2_ Regeneration. ACS Energy Lett..

[93] He G., Li Z., Zhao J., Wang S., Wu H., Guiver M.D., Jiang Z. (2015). Nanostructured Ion-Exchange Membranes for Fuel Cells: Recent Advances and Perspectives. Adv. Mater..

[94] Strathmann H., Krol J., Rapp H.-J., Eigenberger G. (1997). Limiting current density and water dissociation in bipolar membranes. J. Membr. Sci..

[95] Li Y.C., Yan Z., Hitt J., Wycisk R., Pintauro P.N., Mallouk T.E. (2018). Bipolar Membranes Inhibit Product Crossover in CO_2_ Electrolysis Cells. Adv. Sustain. Syst..

[96] Pribyl-Kranewitter B., Beard A., Schuler T., Diklić N., Schmidt T.J., Patru A. (2021). Investigation and Optimisation of Operating Conditions for Low-Temperature CO_2_ Reduction to CO in a Forward-Bias Bipolar-Membrane Electrolyser. J. Electrochem. Soc..

[97] Müller J., Zhegur A., Krewer U., Varcoe J.R., Dekel D.R. (2020). Practical ex-Situ Technique To Measure the Chemical Stability of Anion-Exchange Membranes under Conditions Simulating the Fuel Cell Environment. ACS Mater. Lett..

[98] Inaba M., Kinumoto T., Kiriake M., Umebayashi R., Tasaka A., Ogumi Z. (2006). Gas crossover and membrane degradation in polymer electrolyte fuel cells. Electrochim. Acta.

